# A Self-Reported Survey-Based Evaluation of the Real-World Effectiveness of Non-mRNA COVID-19 Vaccines During the Second Pandemic Wave in India

**DOI:** 10.7759/cureus.95407

**Published:** 2025-10-25

**Authors:** Subeikshanan Venkatesan, Amrutha Bindu Nagella, Sukumar Kalvapudi, Akshat Dutt, Karthik Ajith, Varun Muppidi, Varun Anand, Rishab Belavadi, Samiksha Kumar, Venkatesh S Madhugiri

**Affiliations:** 1 Neurology, University of Florida Health, Gainesville, USA; 2 Rehabilitation Science, University at Buffalo, Buffalo, USA; 3 General Surgery, Wyckoff Heights Medical Center, New York, USA; 4 General Surgery, All India Institute of Medical Sciences, Jodhpur, Jodhpur, IND; 5 Neurology, Christian Medical College, Vellore, IND; 6 Neurosurgery, Jawaharlal Institute of Postgraduate Medical Education and Research, Pondicherry, IND; 7 General Surgery, Mayo Clinic, Phoenix, USA; 8 Acute Medicine, Southampton General Hospital, Southampton, GBR; 9 Radiation Medicine, Roswell Park Comprehensive Cancer Center, Buffalo, USA

**Keywords:** breakthrough infections, covaxin and covishield, covid-19 india, covid-19 vaccine, covid-19 vaccine efficacy

## Abstract

Introduction

Breakthrough infections (BIs), defined as SARS-CoV-2 infections occurring in fully vaccinated individuals, provide crucial insights into vaccine effectiveness. Data on BIs after non-mRNA vaccines remains limited.

Aim

This study aimed to evaluate the incidence, characteristics, and risk factors associated with COVID-19 BIs in individuals vaccinated with Covishield (Serum Institute of India Pvt. Ltd.) and Covaxin (Bharat Biotech) in India.

Methods

A survey was conducted from June 1 to September 15, 2021, via Google Forms, disseminated through social media and email. After excluding duplicates and incomplete responses, 5248 were analyzed. Statistical analyses, including chi-squared tests and logistic regression, assessed the risk factors for BIs and their severity.

Results

Among 5248 respondents, 405 (12.9%) reported BIs. Age >60 years was associated with a higher BI rate (p<0.00001), greater severity (p<0.0001), and higher mortality (p<0.0001). Mortality was higher in men (5.67%) than in women (1.37%; p=0.005). BI rates were similar between Covishield (12.7%) and Covaxin (13.9%) recipients (p=0.4). Longer interdose intervals (5-12 weeks) were associated with lower BI rates (p=0.005). Healthcare workers had higher BI rates (p<0.008) but lower severity (p=0.009) and mortality (0.64% vs. 6.76%; p<0.001). Severe BIs were more common in individuals with comorbidities (p<0.0002). Mortality rate was significantly lower in fully vaccinated individuals (2.29%) compared to incompletely vaccinated individuals (6.22%; p=0.016).

Conclusions

Full vaccination reduced severe disease and mortality. Older age, comorbidities, and occupational exposure were key risk factors. Despite higher exposure, healthcare workers had lower infection severity and mortality. These findings highlight the importance of optimized vaccine dosing, booster doses for high-risk groups, and continued public health measures.

## Introduction

The COVID-19 pandemic exerted an unparalleled global impact and affected every individual in the world. As of June 2024, 776 million confirmed cases of COVID-19 had been reported, with an associated mortality of seven million individuals. One of the major factors that led to the control of the pandemic was the rapid development and global deployment of multiple vaccines against COVID-19 at an unprecedented pace.

India commenced its national vaccination program against COVID-19 on January 16, 2021. By June 2024, over 2.2 billion doses of approved vaccines had been administered. Despite the widespread distribution of vaccines, cases of breakthrough infections (BIs), defined as SARS-CoV-2 infection occurring in fully vaccinated individuals, have been frequently reported [1-3]. BIs are the cornerstone to assessing the real-world effectiveness of vaccines and may also provide valuable insights into cross-reactive immune responses in vaccinated individuals when confronted with emergent viral variants. While numerous studies have documented BIs following mRNA vaccination, data on BIs associated with non-mRNA vaccines remains relatively limited [4-7].

In India, two vaccines were initially deployed in 2021. The first, based on a recombinant replication-deficient chimpanzee adenovirus vector encoding the SARS-CoV-2 Spike (S) glycoprotein, was developed at Oxford University and manufactured by AstraZeneca worldwide and by Serum Institute of India in India (Covishield). The second vaccine was a whole inactivated virus-based COVID-19 vaccine developed by Bharat Biotech in collaboration with the Indian Council of Medical Research-National Institute of Virology (Covaxin) [8,9]. India started vaccinating against SARS-CoV-2 on January 16, 2021, in a phased manner: healthcare workers (HCWs) and other frontline workers initially, elderly people with prespecified comorbidities next, and subsequently the general population.

Comparative evaluations of the efficacy of these vaccines emerged shortly after their introduction. However, early reports on BIs in India were almost exclusively derived from HCW cohorts, which, while convenient to study, do not reflect the broader diversity of the general population. As a result, there remains a limited understanding of BI patterns, risk factors, and outcomes in community-based cohorts that encompass varying ages, occupations, comorbidity profiles, and levels of exposure. Furthermore, the majority of prior studies have relied on laboratory-based immunogenicity assays, which, while rigorous, are limited in their ability to provide rapid and scalable data collection required in the context of a rapidly evolving situation [10-16].

It is crucial to assess BIs within a broader and more heterogeneous population to obtain comprehensive insights into the differential susceptibility to infection across various demographic groups. Additionally, examining BIs allows for an understanding of public attitudes toward vaccination. Studies suggest that individuals who experience BIs may demonstrate reduced compliance with receiving booster doses, potentially undermining public health efforts aimed at sustaining long-term vaccine uptake [17,18]. Given the ongoing evolution of SARS-CoV-2 variants, collecting and disseminating robust data on BIs is imperative. This information is critical not only for shaping future public health strategies but also for determining the necessity of periodic booster vaccinations, similar to the strategy employed for influenza and respiratory syncytial virus (RSV) vaccinations.

The objective of this study was to evaluate the incidence and characteristics of BIs in a diverse cohort of individuals vaccinated with the two predominant vaccines in India, Covishield and Covaxin.

## Materials and methods

Survey design and dissemination

This study is based on a survey (Appendices) that was conducted during the second wave of the COVID-19 pandemic. The survey questionnaire was developed using the Google Forms platform. The target population was selected utilizing a combination of convenience and snowball sampling. The survey link was initially disseminated through various WhatsApp groups, accompanied by a request for recipients to further circulate the link. Additionally, the survey link was posted on relevant Facebook groups and on Instagram. Bulk emails containing the survey link were also sent to lists of email addresses obtained from publicly available online sources.

The survey remained open for 3.5 months, from June 1, 2021, to September 15, 2021. Multiple reminders (more than three) were sent to the same WhatsApp groups, and periodic status updates with the survey link and posters were shared. No incentives were provided for completing the survey. 

Questionnaire description

The survey consisted of 59 elements, structured to allow participants to respond on behalf of themselves (33 questions) or for a family member or friend (27 questions). The actual number of questions encountered by each participant was kept to a minimum by employing a logic tree approach. The survey included statements regarding data confidentiality and informed consent, ensuring voluntary and anonymous participation. Respondents were not required to log into a Google account to submit responses, nor were email addresses collected automatically. However, participants had the option to share their email addresses if they wished to do so. The survey was pretested by several individuals, and their feedback was used to modify the questionnaire before the final version was posted on social media and messenger applications. The longest logic path in the survey consistently took approximately 10 minutes to complete.

Definitions of terms

For purposes of data analysis, we defined the following terms in the current study:

Completely Protected (CP)

Individuals were considered completely protected two weeks after receiving two doses of either COVID-19 vaccine.

Incompletely Protected (IP)

Individuals were considered incompletely protected during the period starting two weeks after receiving the first dose of a vaccine until two weeks after receiving the second dose.

Unprotected

Individuals were considered unprotected up until two weeks after receiving the first dose of any vaccine.

BIs

It is any infection occurring in a completely protected individual.

Infection in an Incompletely Protected Individual (IIP)

It is a COVID-19 infection in an incompletely protected individual (vide supra).

Duration to BI/IIP

It is the time in days from two weeks after the last vaccine dose received to the onset of symptoms confirmed by a diagnosis of COVID-19 infection.

Interdose Interval

It is the number of weeks between receiving the first and second doses of any vaccine.

Clinical grades of infection

We created a composite grading system to assess the severity of infection. This system drew on elements of several other standard scoring systems, but was modified to make it appropriate to apply via an online survey that would be answered by persons with no technical medical knowledge [19,20]: clinical grade 0, asymptomatic individuals with a BI; clinical grade 1, symptomatic individuals with BIs, without severe breathlessness or a measured SpO2 below 93%; and clinical grade 2, individuals with BIs exhibiting severe breathlessness or a measured SpO2 below 93%.

Statistical analysis

Incomplete responses were individually analyzed; usable components of incomplete responses were incorporated into the relevant analyses. Responses that contained no usable data points and those that were duplicated were eliminated from further analysis. Frequencies and proportions were calculated for nominal and ordinal variables, while descriptive statistics were used for continuous data. Comparisons of means were performed using an unpaired t-test, and the chi-squared test was employed for frequency comparisons. A significance level of p<0.01 was set for all statistical tests. All analyses were conducted using IBM SPSS Statistics for Windows, Version 28.0 (IBM Corp., Armonk, New York, United States).

## Results

**Figure 1:**
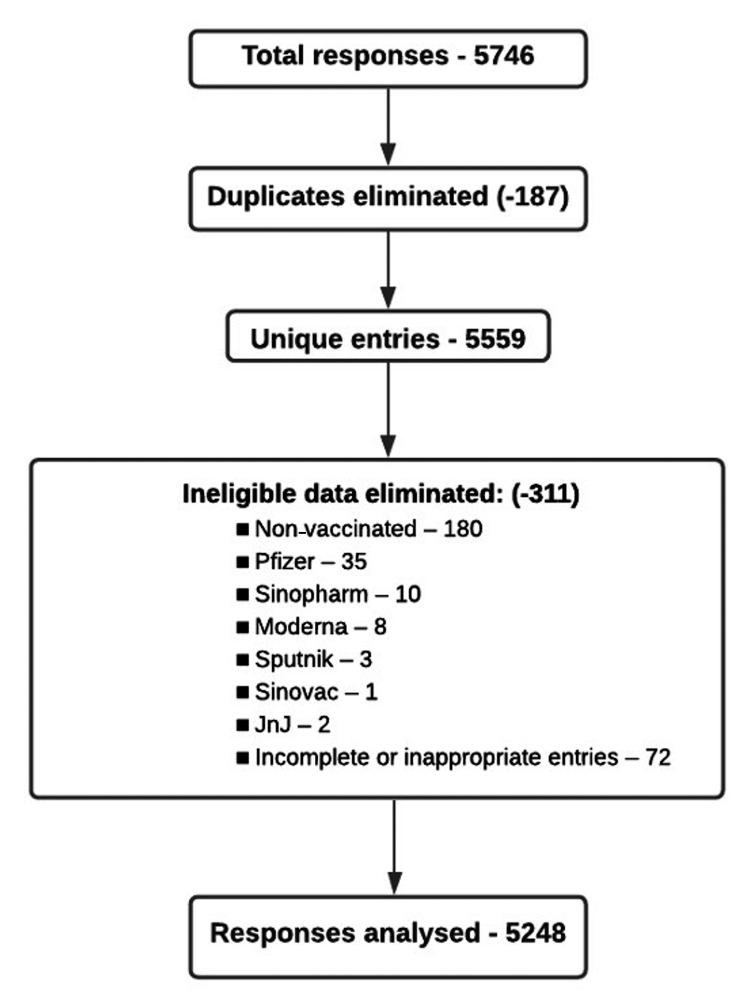
Analysis flowchart showing the number of responses received and the final number of responses included in the analysis

The total number of responses obtained upon the completion of the survey was 5746. After eliminating duplicate entries and ineligible data, 5248 responses were eventually included in the data analysis (Figure 1). Of the respondents, 4307 (82.1%) filled out their own data, whereas 17.9% filled out the survey on behalf of someone else. 

The mean age of survey respondents was 33 years (SD: 5.15; range: 17-95). There were 2099 male (M), 2417 female (F), and three non-binary (NB) respondents. Gender data was not available for 729 respondents. Of the 5248 respondents, 4412 (84%) had received Covishield, and 836 (16%) had received Covaxin. A total of 3131 individuals were completely protected, 1787 were incompletely protected, and 322 were unprotected. 

Among survey respondents, there were 619 (11.8%) self-reported COVID-19 infections diagnosed either by reverse transcription polymerase chain reaction (RT-PCR) assay (576) or computed tomography (CT) of the chest (43). Of the reported infections, 405 (12.9%) were BIs (infections in the completely protected), and 208 (11.6%) infections were reported in incompletely protected respondents (after one dose of the vaccine). No infections were reported in the unprotected category. 

Information about symptoms and disease severity was provided by 396 (203 M, 193 F) respondents with BIs. The distribution of disease severity was as follows: 283 (71.4%) were of grade 0, 75 (18.9%) were of grade 1, and 38 (9.6%) were of grade 2 severity. The mean duration to BI was 55 days (SD: 31; range: 14-195) from the completely protected status. 

BIs and age

**Figure 2:**
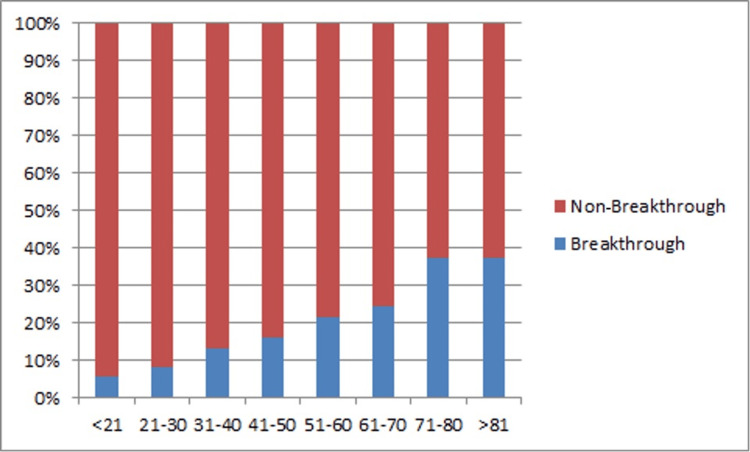
Age distribution of persons who developed breakthrough infections (n=405) and for infections in incompletely protected persons (n=208)

The rate of BI varied significantly with age, with the highest rate of BIs seen in those >60 years of age (Table 1). The differences in the rates of BI between the three age groups were statistically significant (χ²=52.19; V=0.14; p<0.00001). The age-wise distribution of the rate of BIs is shown in Figure 2. 

**Table 1:** Breakthrough infection rates among different categories Χ²: chi-squared statistic; *: healthcare workers who treated COVID-19 patients as outpatients or inpatients

Category	N	Breakthrough	Non-breakthrough	Test statistic	Effect size	Bonferroni adjusted α	P-value
Vaccine type							
Covishield	2579	328 (12.7%)	2251 (87.3%)	χ²=0.47; df=1	Φ=0.012	0.01	0.493
Covaxin	552	77 (13.9%)	475 (56.1%)				
Healthcare worker status							
Healthcare workers*	701	137 (19.5%)	564 (80.4%)	χ²=7; df=1	Φ=0.065	0.01	<0.008
Non-healthcare workers	944	138 (14.6%)	806 (85.4%)				
Interdose interval							
4 weeks	1404	205 (14.6%)	1199 (85.4%)	χ²=8.54; df=3	V=0.052	0.01	0.052
5-8 weeks	1482	172 (11.6%)	1310 (88.4%)				
9-12 weeks	206	20 (9.7%)	186 (90.3%)				
>12 weeks	61	8 (13.1%)	53 (86.9%)				
Age group							
≤30	1596	174 (10.9%)	1422 (89.1%)	χ²= 52.19; df=3	V=0.14	0.01	<0.00001
31-60	899	169 (18.8%)	730 (81.2%)				
>60	245	62 (25.3%)	183 (75%)				

The clinical severity of BIs also differed between different age groups: grade 2 or severe symptoms were reported by 2.8% of those aged less than 30 years, 12.2% of those aged between 31 and 60 years, and 22.2% of those aged more than 60 years (χ²=27.28; V=0.184; p<0.000018) (Table 2). Thus, older persons (age >60) had higher rates of BIs and a higher rate of severe infections. 

**Table 2:** Clinical severity of breakthrough infections among different categories

Category	Grade 0	Grade 1	Grade 2	Test statistic	Effect size	Bonferroni adjusted α	P-value
Vaccine type							
Covishield	239 (70.5%)	65 (19.2%)	35 (10.3%)	χ²=4.107; df=2	V=0.099	0.0083	0.129
Covaxin	49 (63%)	17 (22%)	12 (15%)				
Healthcare worker status							
Healthcare workers	188 (70.7%)	59 (22.2%)	19 (7.1%)	χ²=9.64; df=2	V=0.156	0.0083	<0.009
Non-healthcare workers	95 (73.8%)	16 (12.3%)	19 (14.6%)				
Interdose interval							
4 weeks	143 (70.8%)	40 (19.8%)	19 (9.4%)	χ²=2.879; df=6	V=0.06	0.0083	0.823
5-8 weeks	121 (72.5%)	30 (17.7%)	16 (9.8%)				
9-12 weeks	14 (73.6%)	4 (21.2%)	1 (5.2%)				
>12 weeks	5 (62.5%)	1(12.5%)	2 (25%)				
Age group							
≤30	128 (73.1%)	42 (24%)	5 (2.8%)	χ²=27.28; df=4	V=0.184	0.0083	<0.000018
31-60	121 (74.3%)	22 (13.5%)	20 (12.2%)				
>60	35 (55.5%)	14 (22.2%)	14 (22.2%)				
Recovery characteristics							
Recovered completely	234 (82.7%)	56 (74.7%)	15 (39.5%)	Χ²=56.54; df=6	V=0.268	0.0167	<0.00001
Recovered with persistent symptoms	45 (15.9%)	17 (22.7%)	17 (44.7%)				
Still in hospital at the time of the survey	2 (0.7%)	1 (1.3%)	1 (2.6%)				
Passed away	2 (0.7%)	1 (1.3%)	6 (15.8%)				
Recovery duration							
<7 days	182	33	10	Χ²=51.37; df=6	V=0.254	0.0167	<0.00001
8-14 days	88	31	16				
15-21 days	9	9	7				
>21 days	4	3	6				

BIs among recipients of Covishield and Covaxin

BIs among Covishield and Covaxin recipients were 328 (12.7%) and 77 (13.9%), respectively. The mean (SD) age of Covishield recipients was 33 (15) years, and that of Covaxin recipients was 35 (15) years. There was no significant difference in the number of infections diagnosed among persons who had received one or two doses of Covishield (n=509; 11.5%) and those who had received one or two doses of Covaxin (n=110; 13.2%; c2=1.78; p=0.18). Likewise, there was no significant difference in the number of BIs diagnosed in those completely protected with Covishield (n=328; 12.7%) or Covaxin (n=77; 13.9%; c2=0.6; p=0.4). There was a higher incidence of severe BIs in patients who had received Covaxin (15.07%) than in those who had received Covishield (8.4%); however, this difference was not statistically significant (χ²=3.1; Φ=0.012; p=0.079). 

BIs and interdose interval

The interdose interval was four weeks for 1429 (43.3%), 5-8 weeks for 1524 (46.2%), 9-12 weeks for 258 (7.8%), and >12 weeks for 87 (2.6%) survey respondents. Among completely protected individuals, BIs were seen in 205 (14.6%) with an interdose interval of four weeks, 172 (11.6%) among those with a 5-8-week interval, 20 (9.7%) among those with a 9-12-week interval, and eight (13.1%) among those with a >12-week interval. When the interdose interval was dichotomized into 5-12 weeks or any other interval, there was a significant difference between the rate of BIs between the 5-12-week group (11.4%) and the group with any other interdose interval (14.8%; χ² c2=7.8; V=0.05; p=0.005). The association between interdose interval and clinical severity was not statistically significant (p=0.82). 

BIs among HCWs and non-HCWs

**Figure 3:**
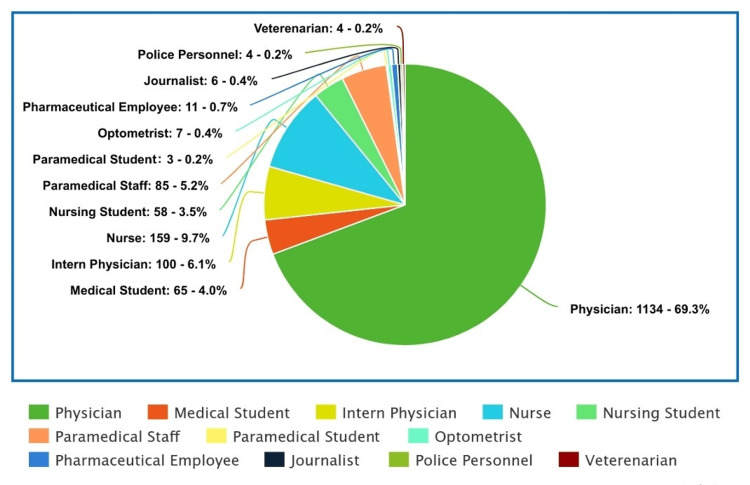
Categories of persons considered as essential workers (N=2271) during the pandemic lockdowns

The survey was answered by 2271 (939 M, 1331 F, 1 NB) essential service providers and 2247 (1159 M, 1086 F, 2 NB) non-essential service providers. The categories of essential and non-essential workers are shown in Figure 3. Of the respondents, 2257 were HCWs (929 M, 1327 F, 1 NB), and the remaining (N=2261) were non-HCWs. The mean (SD) age of HCWs was 30.5 (12.4) years, and that of non-HCWs was 35.6 (17) years. Among the completely protected HCWs (N=1796), 833 (46.4%) did not treat COVID-19 patients and/or treated patients via teleconsultation. Of those, 106 (12.7%) had BIs. One hundred and sixty-six (9.2%) HCWs treated COVID-19 patients on a purely outpatient basis. Of those, 31 (18.6%) had BIs. Five hundred and thirty-five (29.8%) HCWs treated COVID-19 patients on an inpatient basis (including ICUs and ORs). Of those, 106 (19.8%) had BIs. 

Among completely protected non-HCWs (N=944), BIs were seen in 138 (14.6%) (Table 1). The difference in BI rate among HCWs who treated COVID-19 patients (outpatient or inpatient) compared to non-HCWs was statistically significant (χ²=7; df=1; p<0.008).

In the HCW cohort, 188 (70.7%) had grade 0 severity, 59 (22.2%) had grade 1 severity, and 19 (7.1%) had grade 2 severity. Among non-HCWs, 95 (73.8%) had grade 0 severity, 16 (12.3%) had grade 1 severity, and 19 (14.6%) had grade 2 severity. The association between clinical grade and being an HCW was statistically significant (χ²=9.64; df=2; V=0.156; p<0.009). 

The mean age (SD) of HCWs who experienced BIs was 34.1 years (17.1), whereas the mean (SD) age of non-HCWs who experienced BIs was 50.1 years (20). The difference in age between the two groups was highly statistically significant (t=9.7715; 95% CI of the mean difference 18.11 to 14.15; df=403; p<0.0001). 

Clinical characteristics and outcomes

**Figure 4:**
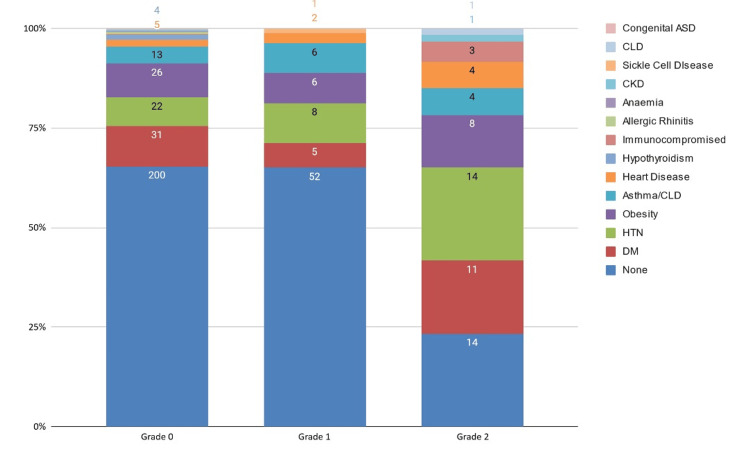
Distribution of comorbidities across the clinical severity grades (total n=386) ASD: autism spectrum disorder; CLD: chronic lung disease; CKD: chronic kidney disease; HTN: hypertension; DM: diabetes mellitus

The most frequently reported symptoms were fever (69.2% respondents), followed by cough (55.1% respondents), loss of smell/taste (52% respondents), and cold/runny nose (52% respondents) (Table 3). Details regarding clinical severity among different survey participant categories can be found in Table 2. Details regarding laboratory findings, imaging findings, management setting, and recovery characteristics of participants with different clinical severity of infections can be found in Tables 4-11. Tables 7-10 display the differences between completely and incompletely protected individuals. Among people with grade 0 infection, 200 (70.7%) had no comorbidities, whereas 83 (29.3%) had at least one comorbidity. Among people with grade 1 infection, 52 (69.3%) had no comorbidities, whereas 13 (30.7%) had at least one comorbidity. Among people with grade 2 infection, 14 (36.8%) had no comorbidities, whereas 24 (63.2%) had at least one comorbidity (Figure 4). The association between clinical grade and having multiple comorbidities status was highly statistically significant (χ²=22.4; df=2; φ=0.267; p<0.0002). 

**Table 3:** Symptomatology of breakthrough infections. 394 participants provided information regarding their symptoms. The percentages in the last column do not add up to 100 because most participants experienced multiple symptoms

Symptoms	Frequency (% of total symptoms)	Percentage of respondents experiencing symptom
Fever	273 (18.3)	69.2
Cough	217 (14.5)	55.1
Cold and runny nose	204 (13.6)	51.7
Loss of smell/taste	203 (13.5)	51.5
Headache	202 (13.5)	51.3
Muscle pain	196 (13.1)	49.7
Diarrhea	67 (4.5)	17
Breathlessness	63 (4.2)	16
Asymptomatic	29 (1.94)	7.36
Sore throat	13 (0.87)	3.3
Fatigue	10 (0.67)	2.53
Conjunctivitis	5 (0.33)	1.3
Malaise	5 (0.33)	1.3
Anorexia	1 (0.07)	0.25
Dizziness	1 (0.07)	0.25
Palpitations	1 (0.07)	0.25
Vomiting	2 (0.13)	0.25
Wheezing	1 (0.07)	0.25

**Table 4:** Clinical grade and imaging features CT: computed tomography

CT status	Grade 0	Grade 1	Grade 2
No CT	183	42	7
Normal CT	52	12	5
Mild disease	42	13	11
Moderate disease	4	7	12
Severe disease	1	0	3

**Table 5:** Clinical grade and blood markers

Blood marker levels	Grade 0	Grade 1	Grade 2
Did not undergo testing	125	26	4
Results reported normal	87	17	7
Results reported mildly elevated	62	25	17
Results reported highly elevated	5	6	9

**Table 6:** Clinical grade and management setting/strategy NIV: non-invasive ventilation; HFNC: high-flow nasal cannula; IMV: invasive mechanical ventilation

Management	Grade 0	Grade 1	Grade 2
Home, without oxygen	240	55	11
Home, with oxygen	0	1	2
Home, with steroids	11	6	1
Hospital, without oxygen	7	2	0
Hospital, with oxygen	0	3	7
Hospital, with steroids	7	5	4
ICU, NIV/HFNC/IMV	1	1	12

**Table 7:** Infections in completely vs. incompletely protected respondents

Protection status	N	Infection	Non-infection	Test statistic (χ²)	Effect size	Bonferroni adjusted α	P-value
Completely protected	3131	405 (12.9%)	2726 (87.1%)	χ²=11.52; df=2	φ=0.047	0.0125	0.0006
Incompletely protected or unprotected	2109	208 (11.6%)	1901 (90.1%)				

**Table 8:** Clinical severity of infections in completely vs. incompletely protected respondents

Protection status	Grade 0	Grade 1	Grade 2	Test statistic	Effect size	Bonferroni adjusted α	P-value
Completely protected	283 (71.5%)	75 (18.9%)	38 (9.5%)	χ²=14.98; df=2	φ=0.195	0.0125	<0.0006
Incompletely protected	110 (55.5%)	57 (28.7%)	31 (15.8%)				

**Table 9:** Healthcare worker status in completely vs. incompletely protected categories

Protection status	N	Healthcare worker	Non-healthcare worker	Test statistic	Effect size	Bonferroni adjusted α	P-value
Completely protected	2230	1796 (79.7%)	461 (10.3%)	χ²=677.03; df=1	φ=0.388	0.0125	<0.0001
Incompletely protected/unprotected	2261	944 (41.8%)	1317 (58.2%)				

**Table 10:** Vaccine type in completely vs. incompletely protected respondents

Protection status	N	Covishield	Covaxin	Test statistic	Effect size	Bonferroni adjusted α	P-value
Completely protected	3131	2579 (82%)	552 (18%)	χ²=16.75; df=1	φ=0.057	0.0125	<0.0005
Incompletely protected/unprotected	2117	1833 (86%)	284 (14%)				

**Table 11:** Duration to infection among different categories The p-values are from a Student's t-test. Bonferroni correction for initial α=0.05.

Category	N	Mean duration (SD), days	Test statistic	Bonferroni adjusted α	P-value
Vaccine type					
Covishield	328	55.4 (29.7)	df=329; t=0.746	0.01	0.493
Covaxin	77	52.3 (34.6)			
Healthcare worker status					
Healthcare workers	243	58.8 (30.4)	df=326; t=3.8609	0.01	<0.0001
Non-healthcare workers	138	44.6 (29.5)			
Interdose interval					
4 weeks	205	61.2 (33.2)	df=3; F=5.96	0.01	<0.001
5-8 weeks	172	47.8 (29)			
9-12 weeks	20	41.2 (30)			
>12 weeks	8	-			
Age group					
≤30	142	55 (30.2)	df=7; F=2.164	0.01	0.036
31-60	136	51 (27)			
>60	50	45.7 (29.8)			
Protection status					
Completely protected	405	54.8 (30.8)	df=493; t=6.8097	0.01	<0.0001
Incompletely protected	208	36.8 (20.8)			

The most frequent medication that the respondents took was ivermectin (49.2% respondents), followed by azithromycin (41.8% respondents), doxycycline (37.8% respondents), and oral corticosteroids (21.1% respondents) (Table 12). 

**Table 12:** Medication history

Drugs used	Frequency	Percentage of total drugs	Percentage of participants who took drugs
Third-generation cephalosporin	1	0.1	0.2
Inhalational steroids	2	0.2	0.4
Antiplatelet	4	0.4	0.8
Monoclonal antibody therapy	5	0.5	1
Hydroxychloroquine	10	0.9	2
Vitamin C/zinc	15	1.4	3
Paracetamol	18	1.7	3.6
Amoxicillin	36	3.4	7.2
Remdesivir	51	4.8	10.2
None	87	8.1	17.5
Favipiravir	93	8.7	18.7
Steroids (oral)	105	9.8	21.1
Doxycycline	188	17.6	37.8
Azithromycin	208	19.5	41.8
Ivermectin	245	22.9	49.2

Of the 619 persons with reported infections, data regarding the final disposition was available for 592 persons. Mortality rates are reported as the ratio of deaths to the number of individuals for whom outcome data were available. The mortality rate in the group that reported COVID-19 infection was 3.55% (n=21). The mortality rate was significantly lower for the fully protected group (2.29%) than for incompletely protected individuals (6.22%; χ²=5.78; p=0.016). 

In the overall cohort, the mortality rate was significantly higher in men (n=17; 5.67%) than in women (n=4; 1.37%; χ²=7.98; p=0.005). In the completely vaccinated group, although the mortality among men (n=7; 3.48%) was still higher than in women (n=2; 1.04%), this difference was not statistically significant (χ²=2.6; p=0.106). The mortality rate was higher in those above 60 years of age (12.5%) than in those below 60 (2.95%; χ²=32.5; p<0.001). In the fully vaccinated group, the mortality in those above 60 was 10%, whereas in the group below 60 years of age, the mortality rate was 1.86% (χ²=20.1; p<0.001). 

The mortality rate was much lower for HCWs (0.64%) than for non-HCWs (6.76%; χ²=16.15; p<0.001). This difference persisted in the completely protected group, with the HCWs having an infection mortality rate of 0.76% vis-à-vis the non-HCW group that had a mortality rate of 5.43% (χ²=8.4; p=0.004). 

The variables that were significantly associated with clinical grade were entered into a main-effects multinomial logistic regression model. Age and comorbid status were the only variables found to predict the clinical severity of BIs (Table 13). 

**Table 13:** Multinomial logistic regression was done to assess the relationship between several predictors and clinical grade, using grade 0 as the reference category For grade 1 versus grade 0, none of the predictors were statistically significant, though healthcare worker status approached significance (B=0.597; p=0.068). For grade 2 versus grade 0, both comorbidity burden and age decade were significant predictors. A higher comorbidity burden was associated with significantly increased odds of being in grade 2 (OR=1.899; 95% CI: 1.326-2.719; p<0.001). Moreover, each additional age decade increased the odds of being in grade 2 by approximately 1.4 times (OR=1.403; 95% CI: 1.087-1.811; p=0.009). HCW: healthcare worker

Clinical grade	Parameter	Std. error	df	Sig.	Exp(B)	95% CI for Exp(B)	
						Lower bound	Upper bound
1	Intercept	0.765	1	0.005	-	-	-
	Comorbidity burden	0.186	1	0.519	1.128	0.783	1.625
	Vaccination status	0.168	1	0.539	1.109	0.798	1.542
	Age decade	0.094	1	0.748	1.031	0.857	1.24
	Interdose interval	0.149	1	0.964	0.993	0.742	1.33
	HCW status	0.327	1	0.068	1.816	0.956	3.448
2	Intercept	1.186	1	0.000	-	-	-
	Comorbidity burden	0.183	1	0.000	1.899	1.326	2.719
	Vaccination status	0.214	1	0.149	1.362	0.895	2.073
	Age decade	0.13	1	0.009	1.403	1.087	1.811
	Interdose interval	0.225	1	0.88	0.967	0.622	1.503
	HCW status	0.451	1	0.439	1.417	0.586	3.426

The variables that were significantly associated with breakthrough status were entered into a backwards elimination binary logistic regression model. Younger age (z=15.19; p<0.0001), being completely immunized (z=4.31; p<0.0001), interdose interval of 5-12 weeks (z=-4.48; p<0.0001), and not being an HCW (z=2.12; p=0.034) all independently predicted a lower chance of being infected. Neither the vaccine received (Covishield vs. Covaxin) nor gender predicted infection status (Table 14).

**Table 14:** Binary logistic regression to identify factors that predicted breakthrough infections

Factors	Odds ratio	Std. error	z	P-value	95% CI	
Vaccine brand	1.04	0.12	0.35	0.726	0.82	1.32
Protection status	1.53	0.15	4.31	<0.0001	1.26	1.86
Interdose interval	0.62	0.07	-4.48	<0.0001	0.49	0.76
Age	1.04	0.002	15.19	<0.0001	1.04	1.05
Gender	0.98	0.09	-0.18	0.86	0.82	1.18
Essential service provider or not	1.24	0.13	2.12	0.034	1.02	1.52
Constant	0.017	0.004	-15.13	<0.0001	0.009	0.03

## Discussion

The present study provides a comprehensive assessment of the real-world efficacy of Covishield and Covaxin and the rate and severity of BIs during the second and third waves of the COVID-19 pandemic in India. Our findings indicate that the overall rate of BIs was 12.9% in fully vaccinated individuals, aligning with previous reports documenting post-vaccination infections. Similar studies have reported BI rates ranging between 5% and 15%, depending on the geographic region, predominant viral strains, and vaccine type [21,22]. The mean duration to BI was 55 days post-vaccination, which could be attributed to factors such as waning immunity, post-vaccination behavior, and viral load exposure. These findings reinforce previous research suggesting that while vaccine-induced immunity is protective, it does not entirely eliminate infection risk, particularly against evolving SARS-CoV-2 variants. 

Despite the occurrence of BIs, most cases were asymptomatic or mild, with only 9.6% classified as grade 2 severity. The incidence of severe infections was significantly lower among fully vaccinated individuals (9.6%) compared to incompletely vaccinated individuals (15.66%; p=0.03). Mortality rates were also notably lower in fully vaccinated individuals (2.29%) than in those incompletely vaccinated (6.22%). These results reaffirm the effectiveness of COVID-19 vaccines in preventing severe disease and reducing hospitalization and mortality rates [8,23,24].

Influence of age and vaccine type on BIs

A significant finding of our study is the higher rate of BIs among older individuals (>60 years), with a statistically significant association between age and infection severity. Our results show that 22.9% of individuals over 60 years who experienced BIs had severe symptoms (grade 2) compared to 2.9% in those under 30 years. This aligns with previous literature indicating that immune responses in elderly individuals tend to be weaker due to immunosenescence, increasing their susceptibility to BIs and severe outcomes [25,26]. Moreover, our logistic regression model identified age as an independent predictor of clinical severity, reinforcing global recommendations for booster doses in older populations and immunocompromised individuals to enhance long-term protection [6].

Comparing Covishield and Covaxin recipients, no statistically significant differences were observed in overall BI rates (12.7% vs. 13.9%; p=0.4). These findings suggest that both vaccines provide similar real-world protection against infection, consistent with previous studies on adenoviral vector-based and inactivated virus-based vaccines [27,28]. Through September 2021, Covaxin accounted for only about one in 10 vaccine doses administered nationwide, largely because its phase 3 trial results were still pending while Covishield already had established efficacy data. In our cohort, however, Covaxin recipients represented 17% of those assessed for BIs, with the remainder having received Covishield. This imbalance, inherent to a survey-based study design, limits the potential for fully matched comparisons between the two groups. Given their different immunogenic mechanisms, further studies on neutralizing antibody titers and cellular immunity in recipients of each vaccine could provide deeper insights into potential variations in efficacy.

Impact of the interdose interval

One notable finding is the significant association between the interdose interval and BI rates. Individuals who received their second dose between four and 12 weeks after the first dose had the lowest breakthrough rate (11.4%) compared to other interdose intervals (14.8%; p=0.005). This is consistent with studies indicating that a longer interdose interval allows better immune priming, resulting in enhanced immune responses post-vaccination. Interestingly, we observed the highest breakthrough rates in the >12-week interdose interval group, which contrasts with phase 3 trial findings that suggested extended intervals (>12 weeks) maximize vaccine efficacy. This discrepancy may reflect the emergence of new viral variants during our study period, or it may be attributable to the relatively small number of respondents in the >12-week category. However, clinical severity did not significantly differ among interdose interval categories [29]. Our study adds to this evidence, further supporting the rationale for maintaining optimal dosing schedules to maximize vaccine efficacy.

Occupational exposure and BIs among HCWs

HCWs had a significantly higher BI rate compared to non-HCWs (p<0.008), particularly among those engaged in inpatient COVID-19 care. Despite high infection rates, mortality in HCWs was significantly lower (0.64%) than in non-HCWs (6.76%; p<0.001), likely due to younger age and fewer comorbidities among HCWs. Increased exposure to high viral loads in clinical settings is a well-documented risk factor for BIs, despite vaccination. Notably, although HCWs and non-HCWs had comparable asymptomatic BI rates, HCWs had nearly twice the rate of mild-to-moderate (grade 1) infections. Conversely, non-HCWs had twice the rate of severe (grade 2) infections, likely due to higher age and comorbidity burden. The latter may be explained by older age and higher comorbidity burden in non-HCWs or due to potential underreporting, reduced routine screening, and differences in access to care. Potential selection bias due to the above factors could not be formally assessed. Nevertheless, these findings underscore the continued need for protective measures, booster vaccinations, and rigorous infection control practices among frontline workers.

Comorbidities, symptomatology, and mortality

Comorbidities emerged as strong predictors of severe BIs. Our data indicate that 63.2% of individuals with grade 2 infections had at least one comorbidity, a statistically significant association (p<0.0002). This aligns with prior research showing that chronic conditions such as diabetes, hypertension, and cardiovascular disease increase the risk of severe COVID-19 outcomes even post-vaccination [30].

The overall mortality rate among infected individuals was 3.55%, with significantly higher mortality in men (5.67%) compared to women (1.37%; p=0.005). Additionally, individuals over 60 years had a significantly higher mortality rate (12.5%) compared to younger individuals (2.95%; p<0.001), consistent with global epidemiological trends. The protective effect of full vaccination was evident, as mortality remained lower in fully vaccinated individuals compared to those partially vaccinated or unvaccinated, emphasizing the critical role of complete vaccination in preventing fatal outcomes.

Implications and future directions

The findings of this study have significant implications for vaccine policy, particularly in optimizing dosing intervals, prioritizing booster doses for high-risk populations, and reinforcing protective measures among HCWs. The data highlight the ongoing need for surveillance of BIs and further research into long-term vaccine-induced immunity. Given the emergence of new SARS-CoV-2 variants, future studies should focus on variant-specific vaccine efficacy and the potential need for adapted vaccine formulations.

Limitations

This study has several limitations. First, as a survey-based study, it is not designed for epidemiological causal inference. Second, the survey's non-random convenience sampling method may introduce selection bias, limiting population-wide generalizability. Survey dissemination via social media platforms and email lists may have introduced sampling bias, potentially restricting our cohort to predominantly internet-literate and urban populations. Despite multiple reminders, non-response bias could not be assessed. Third, the distribution of BIs relative to the interval from vaccination to survey participation was significantly right-skewed (W=0.98; p<0.001). This suggests that participants may have been more likely to recall recent infections than earlier ones, potentially leading to the underestimation of BIs occurring further from vaccination and affecting the accuracy of temporal trend analyses, specifically interdose interval results. Additionally, for fatal cases, reporting bias may have influenced the observed numbers, as deaths could only be captured through proxy reporting by family members. This could have resulted in discrepancies between survey-reported fatality rates and population-level data. Fourth, without knowing which SARS-CoV-2 variants were responsible for individual BIs, we are unable to assess whether differences in variant-specific transmissibility, immune escape, or vaccine effectiveness influenced the observed infection rates. Consequently, our findings may not fully generalize to populations exposed to different variants. Future studies should employ prospective designs and serological assessments to complement self-reported data.

## Conclusions

This study provides valuable real-world insights into the efficacy of Covishield and Covaxin, factors influencing BIs, and their clinical outcomes. While BIs occurred, they were predominantly mild, and full vaccination significantly reduced severe disease and mortality. Older age, comorbidities, and occupational exposure were key risk factors for BIs. These findings reinforce the importance of maintaining optimal vaccine dosing schedules, booster doses for high-risk groups, and continued public health interventions to mitigate the impact of COVID-19.

## Appendices

COVID-19: vaccination status and disease incidence 

Background

The COVID-19 pandemic has affected every human being on the face of this earth. Some of us have contracted the infection. Some of us stayed at home and fought the infection, while some of us had to battle it out in the ICU. Some of us have recovered, while some of us are still dealing with the after-effects of the infection. Some of us have had family members and close friends affected by COVID-19. While most of them have recovered, some have unfortunately lost their lives to this deadly infection.

Context

In this context, it is nothing short of a miracle that in the short span of a year, vaccines against COVID-19 have become available. However, the vaccines are not a panacea, since we have all heard anecdotal reports of breakthrough infections in vaccinated persons. This is a very important event and reflects the real-world efficacy of the vaccines. It becomes even more important to gather this information with the emergence of new virus variants.

Aim

Our aim, through this survey, is to assess the incidence of breakthrough infection and its severity among vaccinated individuals.

Eligibility

If you have received at least one dose of any COVID-19 vaccine, you are eligible to take this survey. IT DOES NOT MATTER whether you have ever had a COVID-19 infection at any time or not. If you have taken even a single dose of any vaccine, you are eligible; please do fill out the survey. Please fill the survey out for YOURSELF first.

NEXT, if you have a family member (or close friend) who was vaccinated (one or two doses) and is unable to take the survey, please do fill in the survey SEPARATELY on their behalf. It DOES NOT MATTER whether they contracted COVID-19 after the vaccination or not; their data is eligible for entry in the survey.

Time Required

It will take 10 minutes or less for you to fill it out.

Data Confidentiality

All data filled in by you will be kept absolutely confidential. Your email ID (if you have chosen to enter it) will be deleted as soon as the survey ends and will not be accessible to us or to anyone else. You will NOT ever be contacted by anyone from the investigator team once you submit the survey. NO individual-level or identifying information will be shared with anyone or even used for analysis. We will only be analyzing anonymized data, and the results of the analyses will be represented as aggregate or group data.

Consent

By answering these questions, you consent to participate in this survey. You also consent to the use of the data you enter for statistical analysis of the results. The results obtained from this survey will be published in a peer-reviewed journal and may be disseminated to other medical professionals, the public at large, and the government. No identifying or individual-level information will be included in these results. The raw data (excluding email IDs) gathered from the survey will be stored by the investigators for at least six years and may be deposited in a data repository in a completely de-identified and anonymized form. You consent to all the preceding when you proceed.

Thank you.

* indicates required question.

Kindly let us know if you are filling in the survey on behalf of someone else.*

Others. I am filling in the details on behalf of a friend/family member.

Self. I am filling in on behalf of myself.

Vaccination status

Kindly provide your email address.

Have you received one/two doses of any vaccine against COVID-19 infection?*

No. I am not vaccinated against COVID-19

Yes. One dose of COVISHIELD

Yes. Two doses of COVISHIELD

Yes. One dose of COVAXIN

Yes. Two doses of COVAXIN

Other:

Vaccination details (one dose)

When did you receive the vaccine?*

Date of receiving the vaccine

Date

Vaccination details (two doses)

When did you receive the last dose of the vaccine?*

Date of receiving the last dose of the vaccine

Date

What was the interval between two doses of the vaccine?*

Breakthrough infection 

Have you contracted the COVID-19 infection after getting the vaccination?*

No

Yes. RT-PCR/CBNAAT/TRUENAAT/rapid antigen test positive

Yes. CT of the chest suggestive of COVID-19

Vaccinated and negative for the infection

Age (in years)*

Gender*

Male

Female

Non-binary

Are you an essential service provider?*

No

Healthcare worker

Police personnel

Municipal corporation worker

Journalist

Other:

Healthcare worker vaccinated and negative for the infection

Please mention whether you're a*

Doctor

Nurse

Paramedical worker

MBBS student

Intern

Other:

Do you treat/interact with COVID-19 patients on a regular basis?*

Yes

No

If yes, in what setting?*

Check all that apply

Outpatient basis

Inpatient: wards

Inpatient: ICUs

Don't treat

OR

Dental hospital

Teleconsultation

Other:

Healthcare worker vaccinated and COVID-19 positive

Please mention whether you're a*

Doctor

Nurse

Paramedical worker

MBBS student

Intern

Dental student or dentist

Other:

Do you treat/interact with COVID-19 patients on a regular basis?*

Yes

No

If yes, in what setting?*

Check all that apply

Outpatient basis

Inpatient: wards

Inpatient: ICUs

Both

Other:

COVID-19 positive

When did you test positive for the COVID-19 infection?*

Date

Age (in years)*

Gender*

Male

Female

Non-binary

Are you an essential service provider?*

No

Healthcare worker

Police personnel

Municipal corporation worker

Journalist

Other:

Were you a PRIMARY contact of a lab tested/CT suggestive of COVID-19-positive patient?*

Yes

No

Don't know

Disease severity 

How symptomatic were you when you had the infection?*

Asymptomatic

Mildly symptomatic

Severely symptomatic

Which of the following symptoms did you experience?

Asymptomatic

Fever

Cough

Breathlessness

Loss of sense of smell/taste

Diarrhea

Muscle pain

Headache

Cold

Other:

Did you experience any of the following symptoms?*

Kindly indicate the symptoms you/your family member experienced

No

High-grade fever (>103)

Severe cough

Severe breathlessness

Fast breathing

Severe diarrhea

Drop in SpO2 <93% as measured with a pulse oximeter

Other:

Do you have any comorbidities/high-risk factors for severe disease?*

None

Diabetes

Hypertension (high BP)

Obesity

Heart disease

Asthma/chronic lung disease

Chronic kidney disease

Chronic liver disease

On chemotherapy

Immunocompromised status

Other:

Did you get a CT of your chest?

No. I did not get a CT chest

Yes. CT was normal

Yes. CT suggestive of mild disease

Yes. CT suggestive of moderate disease

Yes. CT suggestive of severe disease

If you got any blood tests done, then were you told that the markers like CRP or D-dimer or others were deranged?

Markers include ESR, CRP, ferritin, D-dimer, IL-6, LDH, etc.

No. I did not undergo any blood tests

The test results were normal

The markers were mildly elevated

The markers were highly elevated

What treatment was necessary?*

None; managed at home WITHOUT oxygen support

None; managed at home WITH oxygen support

Hospitalization with oxygen support

ICU care with NIV/invasive ventilator support/HFNC

Managed at home with steroids

Hospitalization with steroids to reduce fever

Other:

Did you need any of the following drugs in the management of your infection?

Check all the applicable ones

Remdesivir

Tocilizumab

Steroid (dexamethasone, prednisolone/methylprednisolone)

Plasma therapy

Favipiravir (Fabiflu)

Ivermectin

Hydroxychloroquine

Amoxicillin/AmoxyClav/Augmentin

Doxycycline

Azithromycin

None

Other:

How long did your symptoms last?*

How are you feeling now?*

Vaccination status (filled on behalf of others)

Has he/she received one/two doses of any vaccine against COVID-19 infection?*

No

Yes. One dose of COVISHIELD

Yes. Two doses of COVISHIELD

Yes. One dose of COVAXIN

Yes. Two doses of COVAXIN

Other:

Vaccination details (one dose) (filled on behalf of others)

How many weeks have elapsed since he or she received the first dose of the vaccine?*

Please provide the date of vaccination if you know

Date

Vaccination details (two doses) (filled on behalf of others)

How many weeks back did he/she receive the second dose of the vaccine?*

1

2

>2 weeks

1 month

Other:

Please provide the date of the vaccination if you know

Date

What was the interval between two doses of the vaccine?*

Breakthrough infection (filled on behalf of others)

Has he/she contracted the COVID-19 infection after getting the vaccination?*

No

Yes. RT-PCR/CBNAAT/TRUENAAT/rapid antigen test positive

Yes. CT of the chest suggestive of COVID-19

Vaccinated and COVID-19 positive (filled on behalf of others)

When did he/she test positive for the COVID-19 infection?*

Please provide the date of testing positive if you know

Date

Age of the person (in years)*

Gender*

Male

Female

Non-binary

Is he/she an essential service provider?*

No

Healthcare worker

Police personnel

Municipal corporation worker

Journalist

Other:

Was he/she a PRIMARY contact of a lab tested/CT suggestive of COVID-19-positive patient?*

Yes

No

Don't know

Vaccinated and COVID-19 positive (filled on behalf of others)

Please mention whether he/she is a*

Doctor

Nurse

Paramedical worker

MBBS student

Intern

Other:

Does he/she treat/interact with COVID-19 patients on a regular basis?*

Yes

No

If yes, in what setting?*

Check all that apply

Outpatient basis

Inpatient: wards

Inpatient: ICUs

Other:

Disease severity (filled on behalf of others)

How symptomatic was the person when he/she had the infection?*

Asymptomatic

Mildly symptomatic

Severely symptomatic

Which of the following symptoms did he/she experience?

Asymptomatic

Fever

Cough

Breathlessness

Loss of sense of smell/taste

Diarrhea

Muscle pain

Headache

Upper respiratory infection

Cold and running nose

Other:

Did he/she experience any of the following symptoms?*

Kindly indicate the symptoms you/your family member experienced

No

High-grade fever (>103)

Severe cough

Severe breathlessness

Fast breathing

Severe diarrhea

Drop in SpO2 <93% as measured with a pulse oximeter

Other:

Did the person suffer from any comorbidities/high-risk factors for severe disease?*

None

Diabetes

Hypertension (high BP)

Obesity

Heart disease

Asthma/chronic lung disease

Chronic kidney disease

Chronic liver disease

On chemotherapy

Immunocompromised status

Other:

Did he/she get a CT of the chest?

No. CT was not done

Yes. CT was normal

Yes. CT suggestive of mild disease

Yes. CT suggestive of moderate disease

Yes. CT suggestive of severe disease

Do you know if any blood tests were done and if the results were deranged?

Markers include ESR, CRP, ferritin, D-dimer, IL-6, LDH, etc.

No tests were done

The test results were within normal limits

The markers were mildly elevated

The markers were highly elevated

What treatment was necessary?*

None; managed at home WITHOUT oxygen support

None; managed at home WITH oxygen support

Hospitalization with oxygen support

ICU care with NIV/invasive ventilator support/HFNC

Other:

Did he/she need any of the following drugs in the management of your infection?

Check all the applicable ones

Remdesivir

Tocilizumab

Steroid (dexamethasone, prednisolone/methylprednisolone)

Plasma therapy

Favipiravir (Fabiflu)

Ivermectin

Hydroxychloroquine

Amoxicillin/AmoxyClav/Augmentin

Doxycycline

Azithromycin

Healvit

Colchicine

Other:

How long did the symptoms last?*

Please let us know how he/she is doing now*

End of survey

We are very grateful to you for taking some of your valuable time off to fill out this survey.
